# Current and Developing Liquid Biopsy Techniques for Breast Cancer

**DOI:** 10.3390/cancers14092052

**Published:** 2022-04-19

**Authors:** Hsing-Ju Wu, Pei-Yi Chu

**Affiliations:** 1 Research Assistant Center, Show Chwan Memorial Hospital, Changhua 500, Taiwan; hildawu09@gmail.com; 2 Department of Medical Research, Chang Bing Show Chwan Memorial Hospital, Lukang Town, Changhua 505, Taiwan; 3 Department of Biology, National Changhua University of Education, Changhua 500, Taiwan; 4 Department of Post-Baccalaureate Medicine, College of Medicine, National Chung Hsing University, Taichung 402, Taiwan; 5 Department of Pathology, Show Chwan Memorial Hospital, Changhua 500, Taiwan; 6 School of Medicine, College of Medicine, Fu Jen Catholic University, New Taipei City 242, Taiwan; 7 Department of Health Food, Chung Chou University of Science and Technology, Changhua 510, Taiwan; 8 National Institute of Cancer Research, National Health Research Institutes, Tainan 704, Taiwan

**Keywords:** breast cancer, liquid biopsy, circulating tumor cells, circulating tumor DNA, microRNA, extracellular vesicles

## Abstract

**Simple Summary:**

Breast cancer is the most common cancer and leading cause of death worldwide. Therefore, it is important to diagnose and treat breast cancer early. Current diagnostic methods include mammography and tissue biopsy; however, they have limitations. Liquid biopsy is a less invasive tool for diagnosis. In this review, we summarize and focus on the recent discoveries on liquid biopsy and development of detection techniques.

**Abstract:**

Breast cancer is the most commonly diagnosed cancer and leading cause of cancer mortality among woman worldwide. The techniques of diagnosis, prognosis, and therapy monitoring of breast cancer are critical. Current diagnostic techniques are mammography and tissue biopsy; however, they have limitations. With the development of novel techniques, such as personalized medicine and genetic profiling, liquid biopsy is emerging as the less invasive tool for diagnosing and monitoring breast cancer. Liquid biopsy is performed by sampling biofluids and extracting tumor components, such as circulating tumor cells (CTCs), circulating tumor DNA (ctDNA), cell-free mRNA (cfRNA) and microRNA (miRNA), proteins, and extracellular vehicles (EVs). In this review, we summarize and focus on the recent discoveries of tumor components and biomarkers applied in liquid biopsy and novel development of detection techniques, such as surface-enhanced Raman spectroscopy (SERS) and microfluidic devices.

## 1. Introduction

Breast cancer is the most common female cancer in 2020, with an incidence of estimated 2.3 million, representing 11.7% of total cancer cases in the world, and the leading cause of cancer mortality in women, which was responsible for nearly 685,000 deaths worldwide [1]. In US, the American Cancer Society’s 2022 update estimated that approximately 287,850 new cases of breast cancer will be diagnosed in US women, with an estimated 43,250 deaths [2]. Based on immunohistochemistry classification, breast cancer is classified to five major molecular subtypes: luminal A (estrogen receptor (ER)+, progesterone receptor (PR)+, human epidermal growth factor receptor 2 (HER2)−, Ki-67 low), luminal B HER2− (ER+, PR+, HER2−, Ki-67 high), luminal B HER2+ (ER+, PR+, HER2+, Ki-67 high), HER2 (ER−, PR−, HER2+), and basal-like (triple-negative (TNBC), ER−, PR−, HER2−), which are related to the clinical outcomes [3,4].

Despite advances in diagnosis and treatments for breast cancer, the standard methods have several drawbacks, such as being invasive, expensive, not suitable for all patients, and low sensitivity and specificity [5]. Classical diagnostic/monitoring techniques include imaging (mammography, ultrasound, MRI, CT, PET, and X-ray) and tissue biopsy [6,7]. Mammography can lead to both false-positive and -negative results, unnecessary exposure to radiation, and the excessive use of biopsies, and it may fail to rapidly detect the changes in tumor burden [8]. Particularly, tissue biopsy is an invasive procedure that is neither extensive enough to capture the overall genomic landscape of breast tumors nor applicable for monitoring treatment response [9]. These limitations point to the urgent need for better and novel non-invasive methods for early detection, patient survival prediction, and treatment response monitoring. Recent advances in molecular testing and genomics have led the trend of personalized and precision medicine.

**Figure 1:**
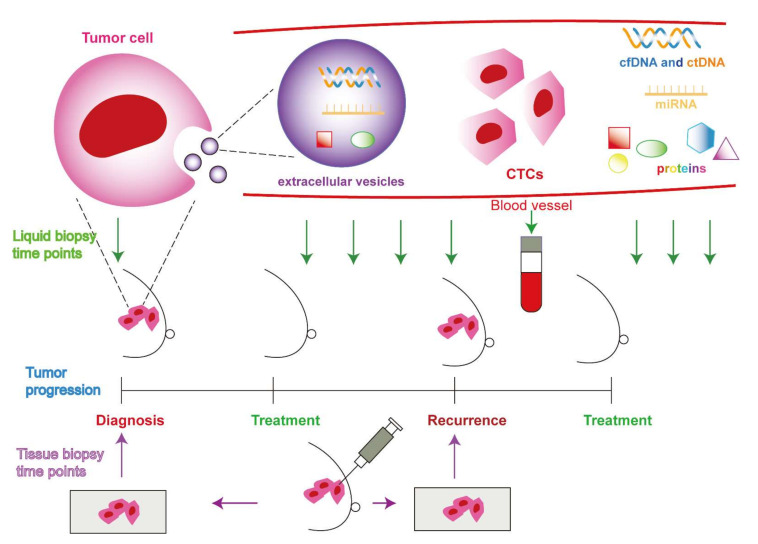
Comparison of liquid biopsy and tissue biopsy. Liquid biopsy is a minimally invasive method and relies on quantification of genetic materials derived from tumor cells and released into circulation, such as circulating tumor cells (CTCs), cell-free DNA (cfDNA)/circulating tumor DNA (ctDNA), circulating tumor RNA, extracellular vesicles (EVs), and circulating tumor proteins. Liquid biopsy allows for early diagnosis and screening, prediction of prognosis, early relapse detection in localized and locally advance breast cancer, minimal residual disease (MRD) identification, and longitudinal monitoring of the disease progression and treatment response. Therefore, liquid biopsy can be applied in as many time points as required during tumor progression and treatments, in order to detect recurrence and monitor response to treatment (green arrows). In contrast, tissue biopsy is an invasive procedure and not applicable for monitoring treatment response; subsequently, tissue biopsy is mainly applied at the time points for diagnosis and detection of recurrence during tumor progression (purple arrows).

Liquid biopsy has attracted considerable attention and become an attractive alternative strategy, as it is a minimally invasive molecular procedure for advanced monitoring of cancer. It relies on the quantification of genetic materials derived from tumor cells and released into circulation, such as circulating tumor cells (CTCs), cell-free DNA (cfDNA)/circulating tumor DNA (ctDNA), circulating tumor RNA, extracellular vesicles (EVs), circulating tumor proteins, and tumor-educated platelets (TEPs) (Figure 1) by collecting body fluids, mostly peripheral blood [10,11]. In comparison with traditional tissue biopsy, liquid biopsy offers a number of notable advantages with easier and non-invasive sampling for serial evaluation [12]. A liquid biopsy, combined with highly sensitive molecular technologies and advance bioinformatics protocols, could reflect the intra-tumoral heterogeneity (spatial heterogeneity) and molecular evolution of a distant metastatic lesion (temporal heterogeneity), which is not possible for conventional tissue biopsies, as the biopsy specimen may not be representative of all the tumor cells [13,14,15,16]. Furthermore, it is possible for the early diagnosis and screening, prediction of prognosis, early relapse detection in localized and locally advance breast cancer, minimal residual disease (MRD) identification, and longitudinal monitoring of the disease progression and treatment response surveillance during adjuvant and neoadjuvant therapies upon sequential sampling, due to its minimally invasive nature [9,17,18] (Figure 1). Despite these, detection limits of liquid biopsy still exist. The low levels of CTCs and ctDNA found in early-stage breast cancer, along with the lack of ctDNA secreting from some tumors, can further complicate detection. Moreover, genetic patterns in primary tumors and metastases vary significantly from patient to patient [12,19]. More sensitive detection methods are urgently needed to improve the clinical application of liquid biopsy.

In this review, we discuss a variety of tumor components and biomarkers applied in liquid biopsy (both already applied in clinical practice and under research), as well as the current developed detection techniques for liquid biopsy in breast cancer, mainly focusing on recent studies.

## 2. Tumor Components

Liquid biopsy components, termed tumor circulome, including CTCs, cfRNA, ctDNA, TEPs, EVs, proteins, and metabolites, are secreted from tumor (apoptotic or necrotic) cells [20,21] (Figure 1). These tumor components present novel and minimally invasive biosources that are clinically implicated in precision medicine [22]. Notably, CTCs and ctDNA have been approved by the US Food and Drug Administration (FDA) as biomarkers in clinical use for cancer management [23].

### 2.1. Circulating Tumor Cells (CTCs)

CTCs are cancer cells compromising of a heterogeneous population with the majority of cells being highly differentiated, while others have stem cell-like properties (CSCs). They are released from primary and metastatic tumors into the circulation by trans-endothelial transition as single cells or clusters. These cells, which are able to adapt and survive by epithelial-to-mesenchymal transition (EMT) in the bloodstream and different tissues, can form new tumors or metastases [24,25,26,27]. Interacting with blood components, such as platelets, is critical for promoting tumor cells for subsequent metastasis [28], and interaction with immune cells results in evasion from immune surveillance and formation of metastases [29,30]. 

There are a considerable number of studies demonstrating CTC detection as an effective technique for the evaluating treatment efficacy, early diagnoses, metastatic progresses, recurrence, and prognosis [31,32,33], and it was correlated with unfavorable prognosis, shorter disease-free survival (DFS) and overall survival (OS), lack of treatment efficacy with poor recurrence-free survival (RFS), and tumor progression [34,35,36,37]. Several researches showed that CTC enumeration could be an independent prognostic tool for early breast cancer patients, particularly for metastatic breast cancer [38,39]. CTCs are substantially less abundant in the blood of patients with early stage of tumors [34,35,36,37]. Cristofallini et al. applied CTCs, detected by CellSearch system, to stratify patients into Stage IV aggressive with ≥5 CTCs/7.5 mL and Stage IV indolent with <5 CTCs/7.5 mL. In a pooled analysis of 2436 metastatic breast cancer patients, Stage IV indolent patients had significantly longer median OS (36.3 months) than Stage IV aggressive patients (16.0 months, *p* < 0.0001), independent of metastasis localization, tumor subtype, and molecular variables [40]. Therefore, this study further demonstrated CTC count is an important prognostic tool for metastatic breast cancer. More recently, 1933 HER2- metastatic breast cancer patients who participated in DETECT III and IV trials were screened, and it was confirmed that the CTC count has a high prognostic relevance [41]. Intriguingly, patients with ER- and PR+ tumors were more likely to harbor ≥1 CTC with strong HER2 staining, and it was significantly associated with shorter OS (median OS: 9.7 vs. 16.5 months in patients with CTCs with negative-to-moderate HER2 staining, *p* = 0.013). This study indicates that CTC detection, in patients with HER2- breast cancer, is a strong prognostic factor, and it remains the largest study conducted in HER2- metastatic breast cancer.

In addition to the prognostic value, in the STIC CTC randomized, multicenter prospective, noninferiority phase 3 trial, 755 hormone receptor (HR)+, HER2- metastatic breast cancer patients were allocated into either clinician-driven group, where the decision to administer hormone therapy or chemotherapy was made clinically without the CTC results, or a CTC-driven group, where endocrine therapy was administered if CTC <5/7.5 mL and chemotherapy administered if CTC ≥5/7.5 mL. Median progression-free survival (PFS) was significantly longer in the CTC-driven arm (15.5 months, 95% CI: 12.7–17.3), compared with the clinically-driven arm (13.9 months, 95% CI: 12.2–16.3) [33]. This key study demonstrated that CTC is promising to direct therapy. However, there is the need for more studies to validate this. Other studies also proved that CTCs can be applied in real-time monitoring treatment responses at different time points during the tumor progression and for the detection of relapses [42,43] (Figure 1). In another study of F.C. Bidard’s group, the CirCe01 trial evaluated the clinical utility of CTC-based monitoring of therapy [44]. In this prospective, multicentre, randomized phase III study (NCT01349842), patients with metastatic breast cancer, scheduled beyond the third line of chemotherapy, were randomized between the CTC-driven arm and standard arm. However, OS was not significantly different between two groups (*p* = 0.8). In subgroup analyses, patients with no CTC response who switched chemotherapy early nevertheless experienced longer median PFS and OS than those who did not.

Beside blood, as the most commonly studied and clinically used fluid in liquid biopsy, Malani et al. [45] recently applied the CTC count in cerebrospinal fluid (CSF) diagnose leptomeningeal metastases in HER2+ breast cancer patients. Their study also proved that CSF CTC enumeration could assess the tumor burden in the central nervous system during therapy for leptomeningeal metastasis and before detectable changes on MRI images or CSF cytology [45]. Importantly, these recent studies on CTC, as a liquid biopsy, confirmed its clinical value in prognosis and role in dynamic and real-time monitoring of treatment, although there is no current clinical application of CTC [39].

### 2.2. Cell-Free DNA (cfDNA) and Circulating Tumor DNA (ctDNA)

Like CTC, cfDNA and ctDNA play important roles in liquid biopsy. cfDNA refers to the double- or single-stranded fragmented DNA liberated into body fluids, such as blood, saliva, lymph, tear fluid, bile, urine, milk, sweat, mucous suspension, amniotic, cerebrospinal and pleural fluids, cervicovaginal secretion, and wound efflux, by both normal and tumor cells, whereas circulating tumor DNA (ctDNA) represents only a fraction of cfDNA derived from the tumor tissue [9,46,47]. Specific patterns of cfDNA can be analyzed ex vivo to characterize the targets of interest [48]. While cfDNA is present in healthy controls, its concentration is significantly lower in healthy subjects, compared to cancer patients, due to active nuclease degradation [49,50]. 

In addition to cfDNA gene sequence and mutation, cfDNA can be further analyzed for epigenetic alterations, such as DNA methylation, histone modifications, and expression of long and micro non-coding RNAs [51,52]. Methylation changes in DNA contribute to gene expression regulation and play a significant role in the etiology of early breast cancer [53,54]. The DNA methylation pattern is retained in the cfDNA released from its tissue origins of tumor cells [55,56]. Therefore, DNA methylation could serve as important biomarkers for diagnosis of cancer [57]. Indeed, DNA methylation has been assessed in cfDNA in several studies, both single and panels of genes have been demonstrated as diagnostic tools [58,59,60]. Furthermore, the methylation patterns of cfDNA could be also related to relapse, metastasis, and survival [5]. Panagopoulou et al. established a cfDNA methylation panel of five cancer-related genes (*KLK10*, *SOX17*, *WNT5A*, *MSH2*, and *GATA3*) and found that increased methylation of three or more and four or more genes (*KLK10*, *SOX17*, *WNT5A*, and *MSH2*) significantly correlated to OS (*p* = 0.042, 0.043, and 0.048) and the absence of pharmacotherapy response (*p* = 0.002), respectively. Subsequently, using machine learning combined clinical data and experimental findings, they developed multi-parametric prognostic signatures for the prediction of survival and treatment response to chemotherapy in metastatic breast cancer [19].

Correlations between elevated concentrations of cfDNA and tumor stage, tumor size, and nodal involvement were demonstrated [19]. In particular, Panagopoulou et al. showed that the metastatic breast cancer patients who had cfDNA levels > median value of 496.5 ng/mL had significantly shortened PFS, compared with those who had < median value of cfDNA (*p* = 0.036), indicating cfDNA quantification could serve as a prognostic marker for PFS. For the predictive value of cfDNA levels for the treatment response of metastatic breast cancer patients to first-line chemotherapy, the median value of cfDNA of the “non-responders” (970.0 ng/mL) was significantly higher than that of the “responders” (465.0 ng/mL, *p* = 0.026), thereby demonstrating cfDNA as a potent predictive marker for response to first-line chemotherapy [19]. The prognosis values of the combination of CTC and cfDNA were firstly evaluated by Ye et al. [61] by collecting blood samples from 117 metastatic breast cancer patients. High levels of CTC (CTC ≥ 5) and cfDNA, individually or jointly, had significantly higher risks of PFS and OS (CTC: *p* < 0.001 for PFS, *p* = 0.001 for OS; cfDNA: *p* = 0.001 for PFS, *p* = 0.002 for OS; joint effect: *p* < 0.001 for PFS, *p* = 0.002 for OS). In a similar result, Fernandez-Garcia et al. compared cfDNA and CTCs with conventional breast cancer blood biomarkers (CA15-3 and alkaline phosphatase (AP)) by analyzing blood samples from 194 metastatic breast cancer patients. Their results showed that both CTCs and total cfDNA levels are predictors for OS (*p* = 0.001 and 0.024, respectively), while only cfDNA correlated with PFS (*p* = 0.042), indicating their potential clinical application of liquid biopsy [62]. 

Generally, ctDNA can be released into the bloodstream by excretion and transport in exosomes or during the apoptosis and necrosis of tumor cells [47]. ctDNA is a small nucleic acid fragment of about 134–144 bp [50,63]. ctDNA is more abundant than CTCs, but it is more rapidly cleared from circulation, within hours, than CTCs. Moreover, ctDNA has been demonstrated to accurately represent the mutational profile of CTCs; Thierry et al. showed that ctDNA can capture the majority of mutations found in tissue biopsy, such as the *PIK3CA* and *ESR1* mutations [64]. However, the evidence on the prognostic value of ctDNA in metastatic breast cancer is rather limited, especially compared with CTCs [65]. Specific somatic DNA mutations, loss of heterogeneity (LOH), and epigenetic alterations, such as methylations, are the valuable factors for precisely discriminating the cfDNA from normal cell and tumor cell [66]. LOH is a cross chromosomal event that results in the loss of one normal allele producing a locus with no normal function [67]. This is a common mechanism for cancer development as the inactivation of a tumor suppressor gene occurs [68]. ctDNA has been demonstrated to detect cancer in early stages [69,70], determine prognosis [13], real-time monitor treatment response [71], and determine therapeutic resistance [72], MRD after primary treatments, and relapse [73,74]. Minimally invasive serial measurement of ctDNA might, thus, monitor and predict treatment response, presenting an advantage over tissue biopsy [5,75,76,77] (Figure 1). Remarkably, increases in ctDNA levels could predict disease progression several months before standard imaging techniques [64]. However, ctDNA has not yet been validated to apply in clinical practice [78].

Prognostically, ctDNA detection was correlated with poor survival in early breast cancer [79,80,81,82]. As in early breast cancer, the quantity of ctDNA is associated with a worse outcome in metastatic breast cancer [75,76,77,83,84,85]. In both the INSPIRE phase II and LOTUS randomized phase II trials, ctDNA levels in TNBC were correlated with PFS, OS, and overall clinical response rate (ORR) [86].

In the aspect of recurrence, a prospective and multicenter study utilized serial plasma samples to assess patients with early-stage breast cancer [74]. Somatic mutations of primary tumors were identified by sequencing, and personalized tumor-specific digital polymerase chain reaction (digital PCR, dPCR) assays were applied to surveil these mutations. Plasma samples were collected every three months for the first-year follow-up and subsequently every six months. The results showed that the presence of ctDNA had a median lead time of 10.7 months before the development of clinical symptoms, indicating ctDNA could predict relapse. Moreover, the use of ctDNA could detect extracranial metastatic relapse in 96% of patients. This addressed that the use of ctDNA, as a surveillance technique, may improve survival. 

A number of studies have evaluated ctDNA levels in both the neoadjuvant and adjuvant therapies [81,82,87,88,89]. In the phase 2 I-SPY 2 trial, Magbanua et al. examined the serial ctDNA test, in early breast cancer patients undertaking neoadjuvant chemotherapy, for predicting pathologic complete response (pCR) and risk of recurrence. Blood samples were collected at several time points, i.e., pretreatment, after therapy initiation, between regimens, or prior to surgery. Patients who remained ctDNA-positive after therapy initiation were significantly more likely to have residual disease after neoadjuvant chemotherapy (83% non-pCR) than those who were ctDNA-negative (52% non-pCR, *p* = 0.012). After neoadjuvant chemotherapy, the presence of ctDNA was associated with lower pCR rates, whereas ctDNA clearance after treatment was correlated with longer survival. Therefore, personalized monitoring of ctDNA during treatment may be a good predictor treatment response [81]. McDonald et al. also demonstrated nonmetastatic breast cancer patients with lower ctDNA concentrations achieve pCR than patients with higher ctDNA level after neoadjuvant therapy (*p* = 0.0057) [89], illustrating that personalized ctDNA panels could monitor breast cancer progression in the neoadjuvant setting. Most recently, Papakonstantinou et al. [90] performed a systematic review and meta-analysis to investigate the prognostic value of ctDNA in patients with early breast cancer treated with neoadjuvant therapy. The association between the detection of ctDNA, both at baseline and after completion of neoadjuvant therapy, and worse relapse-free survival (HR: 4.22, 95% CI: 1.29–13.82 and HR: 5.67, 95% CI: 2.73–11.75, respectively) and OS (HR: 19.1, 95% CI: 6.9–53.04 and HR: 4.00, 95% CI: 1.90–8.42, respectively) were observed, whereas the detection of ctDNA did not achieve a pCR. Therefore, this meta-analysis again supports the previous studies.

In metastatic breast cancer, Darrigues et al. also collected plasma samples of 61 patients at different time points, i.e., before treatment, at day 15, at day 30, and at disease progression, and proved that treatment with palbociclib and fulvestrant can be successfully monitored by serial ctDNA measurements before radiological evaluation [77]. However, more large, prospective, and randomized trials are needed. Interestingly, a study evaluated the predictive and prognostic values of ctDNA in 26 TNBC patients and observed a significant rise in ctDNA levels after neoadjuvant therapy was predictive of residual tumor and, thus, an incomplete pathologic response. This also indicated worse relapse-free survival (*p* = 0.046) and OS (*p* = 0.043) [79]. These studies support using serial monitoring of ctDNA for accurate assessment of tumor progression in real time, resulting therapeutic decision making. However, more clinical studies will be required before ctDNA monitoring can be implemented in a clinical setting [12,79]. 

### 2.3. Non-Coding RNAs

It is known that RNA, especially non-coding RNA (ncRNA), plays significant roles in the deregulation of cell function and cancer development. Like CTC and ctDNA, RNA can also be secreted from tumor cells into blood and other biological fluids of cancer patients and, thus, as a potential analyte in liquid biopsy [91,92]. However, RNA is less stable than CTC and DNA and hindered by the variability in the methodologies performed [93]. Despite these, there are growing evidences depicting the importance of circulating ncRNAs representing 80% of the total circulating RNA application in the field of oncology. They are involved in regulating transduction pathways, acting as tumor activators or suppressors [94]. There are a number of types of ncRNAs, including long non-coding RNAs (lncRNAs), circular RNAs (circRNAs), microRNAs (miRNAs), and PIWI-interacting RNAs (piRNAs) [95,96].

microRNAs (miRNAs) are small ncRNAs (18~25 nt), capable of binding and regulating mRNA expression at the post-transcriptional level [97]. Additionally, miRNAs play important role in cellular communication, proliferation, programmed cell death, and differentiation [98]; thus, they have significant implications in cancer management [99] as potential biomarkers applied in liquid biopsy. miRNAs are derived from CTCs, cell-free miRNAs, apoptotic bodies, or from extracellular vesicles (EVs), either in their lumen or on their surface [100]. miRNAs are the most studied RNA types in tissues and the bloodstream, where several studies proved their clinical application in diagnosis, prognosis, detection of metastasis, and drug resistance [101,102,103,104]. However, little is known about their clinical utility as biomarkers in liquid biopsy, which requires more studies [101].

### 2.4. Extracellular Vesicles (EVs)

EVs refers to the cell-derived membranous vesicles released by all cells into the extracellular environment [105]. They play a role in intracellular communication among tumor cells [106]. EVs carry DNA, mRNA, ncRNA, lipids, metabolites, and proteins protecting and preventing degradation of their cargo from enzymes, such as plasma nucleases, and transferring their contents from a parental to different recipient cells [107,108]. Unlike CTCs, which are mostly released into blood, EVs exist in a variety of body fluids and can be more easily enriched for subsequent analysis than CTCs [109]. cfDNA is secreted into the bloodstream either as free DNA (unbound DNA), bound to protein or lipoprotein complexes (nucleosomes and vitrosomes) [110], or enclosed in EVs [111,112]. 

It has been proven that EVs, involve in the tumor development and initiating the formation of premetastasis niche, play a role in intracellular communication [113]. Tumor-derived vesicles also carry the molecular footprint reflecting the genetic status of parental tumor cells [114]. EVs have been demonstrated as diagnostic, prognostic, and therapeutic agents in clinical settings and have also been associated with drug resistance [115]. As a result, EVs are promising biomarkers in liquid biopsy. However, further studies are required to investigate their clinical validity in breast cancer [5]. 

EVs are generally heterogeneous and classified into microvesicles (MVs, also referred to as ectosomes or microparticles), exosomes, and apoptotic bodies, based on origin and size [116,117]. Apoptotic bodies are the largest vesicles (1~5 μm in diameter) derived from budding of apoptotic cells and usually contain nucleosomes, protecting tumor DNA and RNA from degradation by DNAses and RNAses [118,119]. 

The second largest EVs are microvesicles with large diameters (100–1000 nm) that are actively shed from protuberances in the plasma membrane [120,121]. Tumor-derived microvesicles (TDMs) contain DNA reflecting the genetic status of their original cell [5]; they also carry RNA that can be transferred to recipient cells [114]. It was found that the number of TDMs in the plasma of breast cancer patients was significantly associated to disease stages I-IV (*p* < 0.05 and *p* < 0.0001) [122], indicating a clinical value. 

Exosomes, the best studied EVs, with small diameters (30–150 nm) derived from the endocytic pathway, are secreted upon fusion of multivesicular bodies (MVBs) with the plasma membrane [105,117,121]. Exosomes are secreted by almost all types of cells and can be transferred to recipient cells [123]. They also play critical roles in intercellular communication and can deliver their content to other cells in a paracrine fashion. Importantly, exosomes are also detected in biological fluids, including blood, saliva, urine, breast milk, and cerebrospinal fluid, indicating that they can act as mediators in long distance cellular signaling [124,125,126]. In particular, it has been demonstrated that exosomes contribute to cancer development and metastasis, preparation of the pre-metastatic niche, stem cell stimulation, apoptosis, angiogenesis, immunity, and drug resistance [117,127,128,129]. Tumor-derived exosomes also contain cancer-associated miRNA [130] and proteins [131] that could have diagnostic, prognostic, and therapy monitoring values. Exosomal miRNAs are also associated with tumor aggressiveness [132], angiogenesis [133], metastasis [134], and drug resistance [135] in breast cancer. Remarkably, it has been shown that tumor cells secrete more exosomes than normal cells in response to pathophysiological conditions, such as hypoxia in the tumor microenvironment [129]. Furthermore, exosomes from breast cancer patients contain distinct RNA and protein from healthy donors [136,137]. 

EVs represent one of the latest biomarkers in the liquid biopsy field; thus, the clinical application of EVs is still immature, and no standard detection method exists for breast cancer [23]. More clinical studies are required to confirm the clinical relevance of EVs, such as diagnosis and prognosis, and evaluate the sensitivity and specificity of EVs-based assays. 

## 3. Biomarkers

Biomarkers, including DNA, miRNA, and EV, detected in blood or other body fluid, are important for early diagnosis and prognosis. In addition, the ability to monitor cancer progression and assess response to treatment is important for clinicians to determine the most effective therapy [12]. These can all be performed by liquid biopsy [138]. These are elucidated in detail in the following sections (Table 1); the sensitivities of these methods vary from 19% to 98%, and specificities vary from 64% to 100%.

### 3.1. Gene Mutation

#### 3.1.1. For Diagnosis of Breast Cancer

A number of studies have shown that ctDNA *PIK3CA* mutations can be detected in breast cancer [177,178]. Rodriguez et al. [140] evaluated the utility of ctDNA in the diagnosis of early breast cancer patients by comparing the *PIK3CA* and *TP53* mutations of fresh tissue biopsies and plasma samples using amplicon-based SafeSEQ (Sysmex Inostics) technology and NGS TruSeq custom amplicon low input panel (Illumina). Intriguingly, they found that plasma DNA sequencing permitted the identification of additional *TP53* and *PIK3CA* mutations in ctDNA not detected in tumor biopsy sequencing. Furthermore, ctDNA detection was significantly correlated with younger age (*p* = 0.040), higher tumor grade (*p* = 0.041) and size (*p* = 0.033), immunohistochemical subtype (*p* = 0.025), BIRADS category (*p* = 0.004), and lymph node positivity (*p* < 0.001). This study addresses the fact that ctDNA analysis could be used in diagnosis of early breast cancer and highlights the importance of plasma ctDNA as an accurate alternative to tissue biopsy. More recently, Chin et al. showed the *TP53*, *PIK3CA*, and *AKT1* mutations for ctDNA detection. ctDNA detection rates were 37% and 81% for stage I-III breast cancer and metastatic or recurrent breast cancer, respectively. Additionally, the ctDNA detection rate was correlated with disease stage (*p* = 0.00026), nodal spread (*p* = 0.00649), and distant metastases (*p* = 0.0005) [141].

Other studies have investigated additional mutations in ctDNA as a biomarker for early breast cancer detection. Bartnykaite et al. investigated the association between single nucleotide polymorphisms (SNPs) in MDM2 (rs2279744, rs937283, rs937282) and MDM4 (rs1380576, rs4245739) and breast cancer of the I–II stage. Their results showed that the rs937283 AG, rs937282 CG, rs1380576 CC, and rs4245739 AA genotypes were linked to HR+ breast cancer and suggested they may be useful diagnostic biomarker [142]. 

#### 3.1.2. For Prognosis and Recurrence of Breast Cancer

It is important that early diagnosis of recurrence can decrease the mortality of breast cancer patients greatly. Consequently, there are a number of studies on the evaluation of biomarkers for the prognosis of breast cancer. Here, we only highlight the recent studies.

In a sub-study of the NeoALTTO phase 3 trial, a randomized, neoadjuvant dual HER2-targeted therapy study in early HER2+ breast cancer patients, Rothé and colleagues [144] found that the presence of *PIK3CA* and *TP53* mutations, before neoadjuvant therapy, was correlated with low pCR (*p* = 0.0089). This result indicate that targeted treatment could be planned for patients carrying these mutations. Intriguingly, patients with HER2+ breast cancer and undetectable baseline ctDNA had the highest pCR, indicating the need to apply treatment de-escalation strategies.

Zhang et al. [143] developed a novel methodology to detect ctDNA by designing a panel based on COSMIC data, covering 136 genes and integrating with Breast Imaging Reporting and Data System classification (BI-RADS). Remarkably, the predictive value of this combination improved from 74.2% up to 92%. Thus, ctDNA detection is also a sensitive and specific marker indicating worse prognosis, and the combination of ctDNA with current imaging techniques might be applied to reduce surgical overtreatment.

MRD detection could be critical for assessing therapeutic response and guiding subsequent treatment decisions [78]. Hence, several trials specifically evaluated the value of ctDNA for detecting MRD in the post-neoadjuvant and post-surgery setting [88,179]. Parsons et al. developed an ultrasensitive patient-specific ctDNA panel for tracking up to 488 mutations having 100-fold more sensitivity than droplet dPCR (ddPCR). The clinical sensitivity for detecting MRD was 19% at 1-year postoperatively, which was strongly associated with distant recurrence (HR = 20.8; 95% CI: 7.3–58.9). Importantly, the median lead time from first positive test to recurrence was 18.9 months [88]. These patient-specific ctDNA mutations are attractive biomarkers in liquid biopsy and advance the field of precision medicine. However, it is important to avoid false-negative results, sequencing errors, and artifacts.

In addition, novel immunotherapies have emerged for cancer treatment. In a prospective clinical trial in 72 patients with metastatic breast cancer, no significant association was revealed between PD-L1 tumors and CTC expression. Triple negative (TN) phenotype, number of metastatic treatments, metastatic sites, ≥5 CTCs, and PD-L1(+)-CTCs were shown to be significantly correlated with PFS, whereas tissue PD-L1 expression was not. Therefore, unlike PD-L1(+) tumors, PD-L1 expression in CTCs was associated with survival in metastatic breast cancer, indicating a potential role of PD-L1(+)-CTCs as a stratifying factor for anti-PD-1/PD-L1 treatment for metastatic breast cancer patients [139]. 

#### 3.1.3. For Predicting Treatment Response of Breast Cancer

In evaluating the predictive value of cfDNA in metastatic breast cancer patients, a large, phase III, ongoing PADA1 study analyzed the *ESR1* mutation in 1017 ER+ HER2- patients treated with a first-line aromatase inhibitor plus palbociclib at regular intervals [151]. *ESR1* mutations, detected in cfDNA, were associated with significantly shorter PFS on treatment with aromatase inhibitors + palbociclib than those with wild-type *ESR1* (11.0 vs. 26.7 months). This indicated that the presence of the *ESR1* mutation, at baseline, might promote resistance to aromatase inhibitors plus palbociclib.

The mutations in ctDNA may also be promising biomarkers for assessing treatment response or resistance [180]. For example, *HER2* [82], *ESR1* [181], and *TP53* [182] in ctDNA could be applied to monitor therapy response. The ongoing plasmaMATCH trial, a multicenter, multicohort, phase IIa trial of ctDNA, tested on metastatic breast cancer patients from 18 UK hospitals, assesses the utility of ctDNA in predicting treatment response. A total of 1034 patients were recruited into four treatment cohorts, based on mutations identified in ctDNA—cohort A, with *ESR1* mutations treated with extended dose of fulvestrant; cohort B, with *HER2* mutations received oral neratinib and in case of ER+ breast cancer with a standard-dose of fulvestrant; cohort C, with *AKT1* mutations in ER+ cancer treated with capivasertib + standard-dose of fulvestrant; and corhort D, with *AKT1* mutations in ER- cancer or with PTEN mutations treated with capivasertib [146]. Long-term follow-up is ongoing, but preliminary results have shown positive response in cohorts B and C, with 25% and 22% response rates for neratinib and capivasertib, respectively, demonstrating the ctDNA’s utility to identify targetable mutations, *PIK3CA*, *ESR1*, *HER2*, *PTEN*, and *AKT1* in metastatic patients and enables the selection of mutation-directed therapies (NCT03182634) [146]. 

Additionally, there are several studies that identified the *PIK3CA* mutation in ctDNA for treatment prediction. The phase III SOLAR-1 trial determined *PIK3CA* mutation-status using both tissue samples and ctDNA of 572 ER+ HER2- endocrine pretreated metastatic breast cancer patients. For the patients with ctDNA *PIK3CA* mutations treated with alpelisib and fulvestrant, there was a 45% risk reduction in PFS (11 vs. 5.7 months, HR: 0.65, 95% CI: 0.50–0.85, *p* < 0.001) [147,148]. The recently published paper for this trial showed an improvement of 7.9 months in OS for alpelisib treatment in the *PIK3CA* mutated group, but it was not statistically significant (HR: 0.86, 95%: CI 0.64–1.15, *p* = 0.15) [149]. 

In the retrospective study, Chen et al. revealed a direct correlation between ctDNA profiling, therapeutic response, and disease progression in breast cancer patients [145]. They identified multiple ctDNA mutations in HER2+ and HER2- breast cancer patients that reliably associated with treatment response and drug resistance. The *ERBB2*, *TP53*, *EGFR*, *NF1*, and *SETD2* mutations were identified in HER2+ breast cancer as contributing to trastuzumab resistance, whereas in HER2- breast cancer patients with resistance to chemotherapy, genetic variations in the *TP53*, *PIK3CA*, and DNA damage repair genes were shown. The study also demonstrated that longitudinal ctDNA monitoring provides valuable insights for assessing therapy efficacy and therapeutic resistance. 

The predictive value of ctDNA in drug efficacy was confirmed in the phase I/II BEECH trial, which included patients with ER+ metastatic breast cancer treated with paclitaxel and AKT-inhibitor capivasertib vs. paclitaxel and placebo [83]. Several specific mutations, such as *AKT1*, *PIK3CA*, *ATM*, *TP53*, *ERB2* and *ESR1*, have been detected by ddPCR to evaluate ctDNA changes during the treatment. The median PFS in patients with suppressed ctDNA at 4 weeks and in patients with high ctDNA were 11.1 months and 6.4 months, respectively (*p* < 0.0001).

Apart from the *PIK3CA* and *ESR1* mutations, other gene mutations were found to predict treatment response. In MONALEESA 2-, 3-, and 7-trials, the largest biomarker analysis of any CDK4/6 inhibitor in advanced breast cancer [150], blood samples from 1507 ER+ HER2-metastatic breast cancer patients were analyzed at baseline using NGS with a targeted panel of 557 genes. Gene changes in *FRS2*, *PRKCA*, *MDM2*, *ERB2*, *AKT1*, and *BRCA1/2* were correlated with increased PFS benefit of ribociclib treatment; thus, they are potential biomarkers of response. However, the patients with alterations in *CHD4*, *BCL11B*, *ATM*, or *CDKN2A/2B/2C* genes had little or no benefit from ribociclib, indicating the biomarkers of resistance. 

Therefore, these trials showed encouraging results that cfDNA and ctDNA in liquid biopsy may serve as early predictors for therapeutic response, and there are a number of gene mutations serving as biomarkers for this purpose. 

### 3.2. miRNAs

#### 3.2.1. For Diagnosis of Breast Cancer

Shimomura et al. [152] analyzed the sera of 1280 early breast cancer patients and demonstrated that the combination of five miRNAs (miR-1246, miR-1307-3p, miR-4634, miR-6861-5p, and miR-6875-5p) was able to detect early breast cancer with 97.6% sensitivity and 82.9% specificity. Similarly, Cui et al. [153] reanalyzed the dataset from Shimomura et al. [152] and revealed a panel of three miRNAs, miR-1246, miR-6756-5p, and miR-8073, in order to generate an neural network cascade model can successfully diagnose breast cancer. It showed 97.1% accuracy in 429 breast cancer patients and 895 healthy controls (AUC = 0.971, sensitivity = 96.7%, specificity = 97.2%). Therefore, these studies proved the feasibility of miRNA in liquid biopsy for early breast detection. 

The novel method of machine learning models, incorporating a large set of miRNA expression profiles, have been developed for early detection of five types of cancers including breast cancer. For breast cancer, they demonstrated a 91% sensitivity and 90% specificity. This presents the promising value of liquid biopsy, combined with machine learning, that they are more sensitive even in the early stages of cancer, compared to other diagnostic methods, such as cfDNA diagnostics [183].

These studies confirm the great potential of miRNAs as diagnostic biomarkers for breast cancer. However, there is no best miRNA to be applied in the clinical setting yet. Hence, more accurate and robust studies for miRNA are required. 

#### 3.2.2. For Prognosis of Breast Cancer

miRNAs were also investigated as prognostic biomarkers in early breast cancer. By using Exiqon miRCURY microRNA RT-PCR panels, Huo et al. [154] investigated the expression levels of 11 miRNAs between patients with and without recurrence and identified seven miRNAs, including four upregulated (miR-21-5p, miR-194-5p, miR-205-5p, and miR-375) and three downregulated (miR-376c-3p, miR382-5p, and miR-411-5p) for recurrent patients. This seven-miRNA signature showed a better discriminatory capacity than individual miRNA and could be utilized as prognostic biomarkers for both HR+ and TNBC patients. Madhavan et al. [155] established a prognostic miRNA panel template (PROMPT), including 16 miRNAs, i.e., miR-141, miR-144, miR-193b, miR-200a, miR-200b, miR-200c, miR-203, miR-210, miR-215, miR-365, miR-375, miR-429, miR-486-5p, miR-801, miR-1260, and miR-1274a, correlated with OS and RFS. Therefore, these miRNAs could serve as prognostic biomarkers for metastatic breast cancer, which can assist making the decision of the treatment. Papadaki et al. [18] observed the expression levels of a different set of four miRNAs with an increase in the expression of miR-21 (*p* < 0.001), miR-23b (*p* = 0.028), and miR-200c (*p* < 0.001), as well as a decrease in miR-190 (*p* = 0.0032) that discriminated relapsed from non-relapsed patients. Thus, the combined expression of these four miRNAs could be prognostic biomarkers.

Obviously, there is increasing evidence that the use of miRNAs signatures as prognostic biomarkers is increasing, although no consensus has been reached for using in clinical setting. 

#### 3.2.3. For Predicting Treatment Response of Breast Cancer

Apart from the functions described, miRNAs can also be biomarkers for monitoring response in treatment. Hamam et al. proved miR-125b was correlated with chemotherapeutic resistance [156]. Furthermore, miR-155 levels decreased after surgery and four cycles of chemotherapy in breast cancer patients, indicating miR-155 can possibly monitor treatment response [157]. 

### 3.3. EVs

#### 3.3.1. For Diagnosis of Breast Cancer

There are a number of studies identifying miRNAs derived from EVs for breast cancer detection. A recent study proved that a panel including miR-142-5p, miR-320a, and miR-4433b-5p has clinical value as breast cancer biomarker (AUC of 0.8387, sensitivity of 93.33%, and specificity of 68.75%) [158]. Zou et al. [160] focused on the members from the miR-532-502 cluster with tumor regulation roles. They analyzed the expression patterns of miRNAs in the miR-532-502 cluster in approximately 800 plasma and serum samples from breast cancer patients and healthy controls. Three miRNAs (miR-188-3p, miR-500a-5p, and miR-501-5p) in plasma and five miRNAs (miR-188-3p, miR-501-3p, miR-502-3p, miR-532-3p, and miR-532-5p) in serum were significantly increased in breast cancer patients. More recently, Zou et al. [161] investigated the expression of 12 miRNAs from 32 pairs of serum-derived exosomal samples from breast cancer patients and healthy controls and identified 10 miRNAs, i.e., let-7b-5p, miR-106a-5p, miR-19a-3p, miR-19b-3p, miR-25-3p, miR-425-5p, miR-451a, miR-92a-3p, miR-93-5p, and miR-16-5p, to be upregulated in breast cancer patients than controls. 

Besides blood samples, Hirschfeld et al. analyzed the expression of 13 miRNAs derived from exosomes extracted from urine samples of 69 patients with breast cancer and 40 healthy controls [159]. They identified that a specific panel of four urine exosomal miRNAs, including miR-424, miR-423, miR-660, and let7-i, could be utilized as a highly specific combinatory biomarkers for detecting breast cancer (98.6% sensitivity and 100% specificity). 

In the aspect of lncRNA, Zhong et al. discovered that serum exosomal levels of the lncRNA *H19* were significantly more elevated in patients with breast cancer than healthy controls (*p* < 0.001), indicating as a novel biomarker for the diagnosis of breast cancer [162].

There are already some EV proteins, discovered from a large number of patient samples, showing good discriminative power. Thus, they are potential diagnostic biomarkers for breast cancer [23]. Recently, a prospective clinical pilot study revealed that there were increased levels of exosomal Hsp70 in plasma from breast cancer patients than healthy donors [163]. In addition, claudin-7 and claudin-7/CD81 levels in EVs showed no significant correlation with ER, PR, and HER2 status in breast cancer patients, indicating that claudin-7 might be a universal biomarker for the early diagnosis of breast cancer [164]. Vinik et al. [165] performed proteomic analysis of small EV and identified seven proteins, fibronectin, focal adhesion kinase 1 (FAK), dual-specificity mitogen-activated protein kinase kinase 1, β-Actin, p90RSK_pT573, N-cadherin, and proto-oncogene c-RAF, to discriminate breast cancer patients from healthy individuals (sensitivity: 94%, specificity: 82%), in which FAK and fibronectin revealed high early diagnostic accuracy. Furthermore, Li et al. [166] revealed the epidermal growth factor receptor (EGFR) as a potential biomarker candidate for the early diagnosis of breast cancer, with a sensitivity of >90% and different clinical stages of I–IV, although the AUC was ~0.7. Tian et al. [167] identified that the weighted sum of eight plasma EV protein markers, EV signature, including mucin-1, CA-125, carcinoembryonic antigen, HER2, EGFR, prostate-specific membrane antigen (PSMA), EpCAM, and vascular endothelial growth factor (VEGF) was able to discriminate metastatic breast cancer, non-metastatic breast cancer, and healthy donors, with a high accuracy of 91.1%. 

Other studies proved the higher expression of serum exosomal annexin A2 (AnxA2) in breast cancer patients, compared to non-cancer females (*p* < 0.0001), especially for TNBC, rather than luminal and HER2+ breast cancer. In addition, high expression of exosomal AnxA2 levels in breast cancer was significantly correlated with tumor grade (*p* < 0.0001), poor OS (*p* = 0.0353), and DFS (*p* = 0.0301). Hence, in addition to the diagnostic biomarker, exosomal AnxA2 represents a promising prognostic biomarker and therapeutic target of TNBC [168].

Additionally, EV protein biomarkers from other sources were investigated. Takeuchi et al. [169] successfully applied tears, for the first time, to detect breast cancer-related small EVs and discovered that γ-glutamyltransferase 1, in small EVs from tears, could differentiate between breast cancer patients and healthy donors. Further analysis revealed a significant reduction in postoperative γ-glutamyltransferase 1 signals from tear small EVs of patients with stage I breast cancer, before and after total mastectomy, also revealed a significant reduction in postoperative γ-glutamyltransferase 1 signals. Because the sample size was small, further large studies are required to verify this diagnostic biomarker [23].

#### 3.3.2. For Prognosis of Breast Cancer

In the study of Rodríguez-Martínez et al. [170], the miRNA expression in 53 patients was measured before and after neoadjuvant therapy, showing that levels of circulating exosomal miR-21 and miR-105 were significantly higher in metastatic patients, compared to local ones, as well as controls. In HER2+ cancers, the level of miR-21 decreased after treatment with trastuzumab in a neoadjuvant setting, suggesting that miR-21 levels could be the biomarker for monitoring the treatment response [170].

Most recently, three exosomal miRNAs, miR-30b, miR-328, and miR-423 before neoadjuvant chemotherapy predicted pCR. An increase in miR-127 correlated with pCR in TNBC. After the first neoadjuvant chemotherapy, exo-miR-141 was used to predict pCR, whereas non-pCR was predicted by miR-34a, exo-miR182, and exo-miR-183. The candidate miRNAs were significantly correlated with OS, subtype, and metastasis in breast cancer, indicating their promising role as predictive biomarkers of pCR in liquid biopsy [171]. However, this should be further validated by studies with large cohorts. 

In the aspect of protein, the heat shock protein 70 was significantly upregulated in presurgery plasma small EVs from patients with recurrence [165]. Likewise, Rothammer et al. [172] demonstrated that heat shock protein 70 levels in serum from breast cancer patients who developed recurrence or metastases after radiotherapy were significantly higher than those who remained disease-free (*p* = 0.007). As a result, these confirmed that heat shock protein 70 in EVs could be the prognostic biomarkers for breast cancer. 

#### 3.3.3. For Predicting Treatment Response of Breast Cancer

Most studies investigated exosomal miRNAs, but there are still few studies demonstrating that exosomal mRNAs and lncRNAs might be potential biomarkers [184]. In HR+ advanced breast cancer, upregulated exosomal mRNAs encoding cell cycle-regulated thymidine kinase 1 (TK1) (*p* = 0.01) and cyclin-dependent kinase 9 (CDK9) (*p* = 0.03) correlated with poor clinical response to the CDK4/CDK6 inhibitor palbociclib [173]. Furthermore, it was found that lncRNA *HOTAIR* levels were elevated in circulating exosomes in breast cancer patients than those in healthy controls [174]. High exosomal *HOTAIR* levels were also correlated with poor prognosis; thus, it is the potential diagnostic and prognostic biomarker. 

Keklikoglou et al. revealed increased levels of ANXA6 in plasma EVs, compared with pretreatment levels in five of six breast cancer patients undergoing neo-adjuvant treatment, decreased at the end of therapy with partial or complete remission [175]. This addresses the importance of EV proteins that might have the potential to be used as the biomarker clinically for breast cancer prognosis [23].

### 3.4. Proteins

Although proteins are mostly expressed on CTCs or enclosed in EVs, they can be freely circulated in blood stream. Recently, a novel biomarker, protein cellular communication network factor 1 (CNN1), was analyzed in the plasma of 544 breast cancer patients and 427 healthy controls by ELISA [176]. CCN1, formerly cysteine-rich angiogenic inducer 61, is an extracellular matrix-associated signaling protein of the CCN family and can regulate a broad range of cellular functions, such as cell adhesion, migration, and differentiation, by interacting with cell surface integrin receptors [185,186]. Importantly, CCN1 has been involved in breast cancer progression [176]. Remarkably, it showed the cancer detection specificity of 99.0% and sensitivity of 80.0%. Even 81.5% of small T1 cancers were CCN1+. These demonstrated that circulating protein CCN1 could be applied in the early detection of breast cancer, and it was suggested it could be, thus, included in liquid biopsy panels containing other DNA or proteins [176].

## 4. Detection Techniques

The detection methods for tumor components are well-established. There are three main types of detection methods: PCR-based techniques, targeted deep sequencing, and whole-genome sequencing (WGS) [12]. For PCR-based method, dPCR are extremely sensitive and can detect point mutations as low as 0.01% [187]; this is suitable for liquid biopsy as the concentrations of tumor components are always low. Furthermore, dPCR has been developed to ddPCR [188]. However, these techniques require prior mutational information of tumor cells [12]. Unknown mutations are screened by targeted DNA sequencing techniques, cancer personalized profiling by deep sequencing (CAPP-Seq), tagged-amplicon deep sequencing (Tam-Seq), the safe sequencing system (Safe-SeqS), and amplicon sequencing (AmpliSeq) by means of next-generation sequencing (NGS) [189]. Whole-genome (or exome) sequencing (WGS/WES) can provide a more comprehensive ctDNA profile, based on the somatic chromosomal aberrations, copy number variations, and detection of rearrangements; however, they have the disadvantage of decreased analytical sensitivity [15]. The recent development of detection techniques is elucidated in the details in the following section and Table 2.

### 4.1. Detection for CTCs

There is a variety of technologies for detecting CTC. The technique developed by Menarini Silicon Biosystems called CellSearch^®^ is the gold standard and only technique approved by the FDA for isolating and detecting CTCs in metastatic breast, colon, and prostate cancers. This technique target the epithelial marker protein, EpCAM, on surface of CTCs. However, when CTCs do not express EpCAM in the status of EMT or the stem cell stage, they will not be detected. Several studies have confirmed that a count of ≥1 CTCs, in 7.5 mL of blood, by the CellSearch^®^ system, at different time points, is correlated with worse PFS and OS, and recurrence [37,190,207]. Adnatest (QIAGEN^®^) is another commercial technique for CTCs analysis, which uses a combination of antibodies conjugated with magnetic beads for targeting epithelial markers and RT-PCR for detecting mRNAs biomarkers. Kasimir-Bauer et al. [191] utilized this technique to isolate CTCs and demonstrated the correlation between CTC and worse prognosis. In the study by Kwan et al. [192], a digital RNA signature and a technique called CTC-iChip were performed for CTC isolation and detection in early and metastatic breast cancer patients.

In addition, a nanotube-CTC-chip, a newly developed methodology to detect CTCs in early breast cancer patients, was used [193,194]. This technique utilizes label-free nanotube-antibody microarrays using breast cancer-specific antibodies, such as anti-EpCAM and anti-HER2. Remarkably, this technology was able to identify CTCs in the 100% of the studied breast cancer blood samples. Most recently, Abdulla et al. [195] developed a novel antibody functionalized microfluidic (AFM) chip for detection of CTCs in breast cancer patients’ whole blood. AFM chip can achieve capture efficiency of 99.5% and capture EpCAM, CK19, CD45, and DAPI rapidly, demonstrating AFM chip could be beneficial in clinical setting.

### 4.2. Detection for cfDNA 

The targeted DNA sequencing techniques are very useful for analyzing a limited panel of potential mutations in biopsy samples [196,208]. The Oncomine Breast cfDNA (Thermofisher, Waltham, MA, USA) test, based on AmpliSeq technology, is applied in clinical practice to detect mutations in a limited number of genes from breast cancer patients [196]. More recently, Xie et al. [197] validated the newly developed dPCR detection on HER2 status of cfDNA in stage III/IV breast cancer by comparing with tissue biopsy by using immunohistochemistry (IHC)/fluorescence in situ hybridization (FISH). The sensitivity and specificity between dPCR in plasma and IHC/FISH in tissues were 43.75% and 84.38%, respectively, for 224 breast cancer patients. The overall concordance is 66.97%. Therefore, dPCR could be used as a companion diagnostic tool to detect plasma HER2 status. 

### 4.3. Detection for ctDNA 

Generally, there are two main techniques for detecting ctDNA, targeted and untargeted techniques. The targeted technique, referring to ddPCR or beads amplification magnetics PCR (BEAMing-PCR), is applied to detect previously determined tumor-specific mutations, such as *PIK3CA* and *ESR1*. The untargeted technique, on the other hand, refers to digital NGS (dNGS), such as genome-wide analysis of copy number aberrations (CNAs), WGS, WES, or array comparative genomic hybridization (CGH). These methods allow sequencing of large DNA or RNA fragments and are able to detect previously unknown genetic mutations [22]. Measuring ctDNA is more specific than measuring cfDNA; thus, developing tools for ctDNA detection are more clinically applicable [184].

#### 4.3.1. ddPCR

Both ddPCR and BEAMing have been shown to be able to detect *PIK3CA* mutations in plasma ctDNA from patients with breast cancer [199,200]. The Therascreen *PIK3CA* RGQ PCR kit, detecting *PIK3CA* mutations on ctDNA, has been approved by the FDA. It is able to detect 11 mutations in the *PIK3CA* gene from patients with HR+, HER2-, and advanced or metastatic breast cancer. The presence of *PIK3CA* mutations is correlated, with response to treatment with PIQRAY^®^ (alpelisib) [147]. Therefore, this kit may assist doctors stratify breast cancer patients who should be treated with PIQRAY^®^. 

Recently, Wan et al. developed INtegration of VAriant Reads (INVAR), a novel technique for ctDNA detection. INVAR can detect as little as one mutant molecule per 100,000, thus significantly increasing the sensitivity. However, the median integrated mutant allele fraction (IMAF), obtained in early breast cancer, was 5.2 parts-per-million (ppm), much lower than that obtained in advanced melanoma with the 15,000 ppm, addressing the difficulties to detect ctDNA in localized breast tumors [202].

#### 4.3.2. NGS

The first custom-built NGS-based ctDNA test, Signatera™, was launched in 2019, aiming to improve the detection of MRD after surgery, as well as earlier detection of recurrence. Signatera™ provides each individual with a customized blood test, tailored to match the clonal mutations by WES. Coombes et al. validated the clinical utility of Signatera™ on breast cancer patient samples [73]. Coombes et al. [73] performed WES of tumor tissues from 49 patients and designed personalized profiling targeting 16 patient-specific variants, targeted by multiplex sequencing of plasma ctDNA in the detection for recurrence. Importantly, ctDNA was detected in disease relapse in 89% of the relapsed patients, up to two years earlier than imaging with a specificity of 100%. Such early identification of relapse may provide more effective treatments.

McDonald et al. developed a targeted digital sequencing (TARDIS) of ctDNA might be a highly sensitive technique for predicting pCR to neoadjuvant treatment in early and locally advanced breast cancers [89]. TARDIS uses simultaneous deep sequencing of patient-specific panels, comprised of multiple tumor mutations as ctDNA biomarkers for monitoring disease in the pre-surgery setting, as well as to detect MRD [89]. By applying this sensitive technology, McDonald et al. analyzed plasma for personal mutations before neoadjuvant treatment and detected ctDNA in 100% of the samples. ctDNA was then monitored at different time points of neoadjuvant treatment and, expectedly, ctDNA levels were higher in patients with the residual disease, compared to those that displayed pCR. Therefore, TARDIS is a promising test demonstrating the ctDNA clinical relevance as a biomarker for NAT treatment response and MRD detection and surveillance in early breast cancer.

Most recently, *PIK3CA* hotspot mutations in HR+ metastatic breast cancer were analyzed by a newly developed and high-resolution SiMSen-Seq assay [201]. SiMSen-seq, a simple, multiplexed, PCR-based barcoding of DNA and sequencing, allows detection of extremely rare variant alleles at <0.1% frequency [209]. As a result, *PIK3CA* mutations were detected in 47.3% of plasma samples, with identical *PIK3CA* mutation detected in both tissue and plasma in 33.3% patients [201]. This implies detection of *PIK3CA* mutations in plasma using SiMSen-seq is feasible and shows concordance with tissue biopsy.

Both PCR and NGS-based techniques showed promising results; these might be the future trend in clinical practice. 

### 4.4. Detection for miRNA

The current methods for detecting miRNA are reverse transcription-quantitative PCR (RT-qPCR), dPCR, microarray, and NGS. RT-qPCR is the gold standard method for quantifying small amounts of miRNA, showing good sensitivity, reproducibility, and accuracy. dPCR offer another technique for quantifying miRNA [210]. Microarray and NGS approaches are usually utilized for initial screening and obtain profiles of miRNAs, whereas RT-qPCR and dPCR are applied to validate previous results [211].

A newly developed technique for detecting miRNA is called surface-enhanced Raman spectroscopy (SERS) with seed-mediated grown Ag nanopillars (SMGAPs). The electrochemical reduction on the pre-distributed 40 nm gold nanoparticle seeds (sGNP) served as scaffolds for growth of silver ion, and a nanopillar-shaped silver structure was successfully grown on the substrate surface of gold. miR-21 and miR-155 were applied as the SERS diagnostic target. The limits of detection of each labeled target were 451 zmol and 1.65 amol, respectively. Hence, quantitative analysis of miRNA in urine was successful, compared to that of the healthy group [203].

### 4.5. Detection for Protein

Currently, commercialized EV ELISA kits have been available for quantifying common EV proteins, such as the tetraspanins CD63, CD9, or CD81. Mass spectrometry, on the other hand, is the core technique for characterizing protein. Recently, new techniques have been developed for quantifying proteins, such as microfluidics, SERS, high-resolution flow cytometry, antibody microarrays, electrochemical sensors, and DNA aptamers [212]. Furthermore, a localized fluorescent imaging method, termed digital profiling of proteins on individual EV (DPPIE), was recently developed for analysis of multiple proteins, CD63, EpCAM, and mucin-1, on individual EV [204]. High-dimensional data collected from each individual EV would provide more precise information than ELISA. The proportion of CD63/EpCAM/mucin-1 vesicle in patients with breast cancer was significantly higher than that of healthy control with overall accuracy of 91%. Additionally, high-resolution flow cytometry has been commercialized, and the standardization of assay is also being established [205]. With advance in nanotechnology, microfluidic devices are expected to gain better specificity and sensitivity [206]. Moreover, thermophoretic aptasensor (TAS) is rapid, sensitive, and low-cost to profile cancer-associated protein profiles of plasma EVs. This aptasensor method has several advantages that preseparation of EVs is not needed, the total detection time is short (within 3 h), and it has a low cost (less than $1) [167].This work addresses the promising clinical utility of EVs in the care of metastatic breast cancer. Combination of these diverse detection techniques on a microfluidic platform could achieve personalized assistance for the clinical application of EVs in breast cancer [213].

## 5. Current Challenges with Liquid Biopsy

Although liquid biopsy has many advantages over tissue biopsy, there are challenges for liquid biopsy that need to be solved before applying in clinical practice. The challenges for CTCs are isolation, detection limit and feasibility in clinical application. The number of CTCs in blood is low and profoundly diluted by blood cells, making their detection technically difficult particularly in early breast cancer [101,214]. Currently, the CellSearch^®^ platform is the only technique approved by the FDA for isolating CTCs [215]. The isolation of CTCs by CellSearch^®^ platform is based on the expression of EpCAM. However, EpCAM expression is downregulated in most aggressive cancer cells undergoing EMT [216,217] mentioned earlier in Section 4.1. Therefore, this makes CellSearch^®^ platform face the serious limitation of leaving most CTCs undetected in advanced stage of breast cancer [218]. In addition, current biological techniques will inactivate CTCs, which greatly affects the application of CTCs in clinical setting. Thus, there is an urgent need to develop a mild and specific technique for isolating CTCs in vitro.

The difficulty for cfDNA detection is again its low amount in blood [219]. Importantly, the major challenge of utilizing cfDNA for diagnosing breast cancer is the need for a prior knowledge of tumor-specific variants [220]. An impediment of technical detection of ctDNA in early cancer stages is low concentration of ctDNA, relative to the total concentration of cfDNA [15,221]. Early stages of cancers have about <0.1% ctDNA (10 ctDNA copies per 5 mL), in contrast to ~1% in non-metastatic advanced cancer patients and in stage IV 100–1000 copies per 5 mL (up to 10%) were detected [222,223]. Consequently, technical detection of ctDNA in early cancer stages is strongly challenging, due to its extremely low concentration and requires ultra-sensitive technologies. Recently, Stetson et al. compared four commercial NGS gene panel assays for detecting mutations in ctDNAs. However, there were substantial variability among the ctDNA assays, with a range of sensitivity (38~89%) and positive predictive value (36~80%), particularly in the detection of allele frequency variants <1% [224]. These findings indicate that most NGS assay discordance is a result of technical variations; therefore, standardization of sample collection and analysis is urgently needed before ctDNA tests could be implemented in the clinical setting. Lastly, the utility of ctDNA for diagnosis is further complicated by false-positive readouts caused by clonal hematopoiesis of indeterminant potential (CHIP) mutations, which are somatic mutations in blood stem cells in healthy and elderly population [225,226]. CHIP mutations are considered background noise in liquid biopsy samples, and thus they can lead to in accurate diagnosis and subsequently inappropriate therapeutic treatment [227]. Therefore, it is significantly required to develop strategies for accurately identifying CHIP mutations to avoid false positives.

There are challenges in translating an accurate and reliable panel of circulating miRNA to clinical setting, due to their low amount, differences in the cohort size, collection, type of sample and processing, and current inability to detect novel miRNAs [156,210]. There is not a significant overlap in the miRNA panels across the different studies, reflecting the complicated miRNA expression in breast cancer patients [214]. Another variation is the isolation method different for miRNAs from different sources, such as EVs or cancer cells [210].

For exosome, it is required to standardize exosome extraction, which can exclude contaminants, such as lipoprotein particles and protein complexes [228]. Furthermore, the methods need to be fast and convenient for application in clinical practice; however, the current isolation method for exosome is ultracentrifugation, which is tedious [184].

## 6. Conclusions and Future Directions

Despite the recent advance in diagnosis and treatment for breast cancer, breast cancer is still the leading cause of death in women worldwide. Therefore, developing innovative technologies with the clinical potential to detect breast cancer at its early stage and predict treatment response is still highly required. Liquid biopsy has gained much attention as a non-invasive methodology, which serves to obtain key tumor information via blood-based biomarkers for cancer diagnosis and treatment monitoring. All the tumor components of liquid biopsy, including CTCs, cfDNA, ctDNA, miRNA, and EVs, have promising value in diagnosis, prognosis, and treatment prediction. Especially, the greatest advantage of liquid biopsy over tissue biopsy is the “real-time” longitudinal monitoring of disease progression and treatment response. Currently, only a liquid biopsy blood test, for detecting the *PIK3CA* mutation, is approved by the FDA for breast cancer that can be used in clinical practice. Most biomarkers described in this review are still under clinical trials or pilot studies (Table 1).

Despite the benefits mentioned above, the clinical application of tumor components in liquid biopsy remains to be fully established and requires performing multicentre clinical studies with a large cohort of breast cancer patients. Another major problem hampering their application in clinical practice is the lack of a standard procedure. Furthermore, it is difficult to detect circulating tumor in the early stage of breast cancer, as their amounts in the biofluids from patients are very low. Important advances in detection were developed, such as NGS techniques, which can detect ultra-low diluted tumor materials. Once standard and feasible methods are established, these technologies benefit both the patients and clinicians, as they are relatively inexpensive and noninvasive for diagnosing and monitoring early-stage cancers.

Most RNAs show promising utility in liquid biopsy are miRNAs. More characterization of other circulating RNA types, such as lncRNAs, will provide more options for liquid biopsy.

Finally, the combination of different tumor components, i.e., multi-omics approaches, should be considered, in order to fulfil the unmet need in clinical practice.

## Figures and Tables

**Table 1:** Biomarkers utilized for liquid biopsy.

Biomarkers	Clinical Outcome	Sensitivity and Specificity	Clinical Trials	References
CTC				
For Prognosis				
PD-L1 expression in CTCs	PD-L1 expression in CTCs correlates with survival in metastatic breast cancer	-	A total of 72 patients with metastatic breast cancer (prospective clinical trial (NCT02866149))	[139]
cfDNA/ctDNA				
For diagnosis				
ctDNA: *PIK3CA* and *TP53*	Correlation between ctDNA detection with age, tumor grade and size, immunohistochemical subtype, BIRADS category, and lymph node positivity	-	A total of 29 patients	[140]
ctDNA: the *TP53*, *PIK3CA*, and *AKT1*	For the detection of early and advanced breast cancer	ctDNA detection rates: 37% for local or locally advanced breast cancer; 81% for metastatic or recurrent breast cancer	A total of 109 early and metastatic breast cancer patients	[141]
ctDNA: SNPs in MDM2 and MDM4	For the detection of early breast cancer	-	A total of 100 unrelated Lithuanian women	[142]
For prognosis				
ctDNA: a panel, based on COSMIC data, covering 136 genes	Served as a predictor of worse prognosis	Predictive value: 92%	A total of 861 serial plasma and matched tissue specimens from 102 patients with early-stage breast cancer who need chemotherapy and 50 individuals with benign breast tumors	[143]
ctDNA: *PIK3CA* and *TP53*	Absence of detectable *PIK3CA* and *TP53* variants before neoadjuvant therapy was associated with high pCR rates	-	A total of 455 patients (sub-study of the NeoALTTO phase 3 trial)	[144]
ctDNA panel: 488 mutations	Detecting MRD at 1-year postoperatively, which was positively associated with distant recurrence	Sensitivity: 19%	A total of 6 patients with ER+/HER2- metastatic breast cancer and 142 patients with stage 0 to III breast cancer	[88]
ctDNA: *TP53*, *PIK3CA*, and DNA damage repair genes	Correlation between ctDNA profiling and therapeutic response and disease progression	-	A total of 19 HER2+ and 12 HER2- breast cancer patients	[145]
For predicting treatment response				
ctDNA: the *PIK3CA*, *ESR1*, *HER2*, *PTEN*, *and AKT1*	Enables the selection of mutation-directed therapies	Sensitivity: 93%	A total of 1034 patients (plasmaMATCH trial)	[146]
ctDNA: *PIK3CA*	Treatment with alpelisib-fulvestrant prolonged progression-free survival among patients with *PIK3CA*-mutated, HR+, HER2- advanced breast cancer	-	A total of 572 patients (341 patients with confirmed tumor-tissue PIK3CA mutations, SOLAR-1 trial)	[147,148,149]
ctDNA: *AKT1*, *PIK3CA*, *ATM*, *TP53*, *ERB2*, and *ESR1*	Predict PFS in the treatment of paclitaxel and capivasertib	-	A total of 66 patients with ER+ metastatic breast cancer (phase I/II BEECH trial)	[83]
ctDNA: *FRS2*, *PRKCA*, *MDM2*, *ERB2*, *AKT1*, and *BRCA1/2*	Predicted a trend for increased PFS benefit of ribociclib treatment	-	A total of 1507 ER+ HER2- metastatic breast cancer patients (MONALEESA 2-, 3-, and 7-trials)	[150]
ctDNA: *ESR1*	*ESR1* mutations predicted significantly shorter PFS on treatment with aromatase inhibitors and palbociclib	-	A total of 1017 ER+ HER2- patients (a large phase III PADA1 study)	[151]
cfDNA/ctDNA Methylation				
For diagnosis				
*APC*, *FOXA1*, and *RASSF1A*	Methylation levels differed markedly in breast cancer patients in comparison to healthy controls	Sensitivity: 81,82% Specificity: 76,92%	A total of 137 cases of primary breast cancer tissues and 44 cases of plasma samples	[60]
For prognosis				
cfDNA methylation panel of five genes (*KLK10*, *SOX17*, *WNT5A*, *MSH2*, and *GATA3*)	Methylation of ≥3 and ≥4 genes correlated to OS and no pharmacotherapy response, respectively	Sensitivity: 80% specificity: 59%	A total of 150 and 16 breast cancer patients under adjuvant and neoadjuvant therapy, respectively, 34 patients with metastatic disease and 35 healthy volunteers	[19]
miRNA				
For diagnosis				
Combination of miR-1246, miR-1307-3p, miR-4634, miR-6861-5p, and miR-6875-5p	Detect early breast cancer	Sensitivity: 97.6% Specificity: 82.9%	The serum of 1280 patients with early breast cancer	[152]
miR-1246, miR-6756-5p, and miR-8073	For detection of breast cancer	Sensitivity: 96.7% Specificity: 97.2%	A total of 429 breast cancer patients and 895 healthy controls	[153]
For prognosis				
miR-21-5p, miR-194-5p, miR-205-5p, miR-375, miR-376c-3p, miR382-5p, and miR-411-5p	Could be used as recurrence biomarkers for both hormonal positive and TNBC patients	Sensitivity: 92.9% Specificity: 77.4%	A total of 48 breast cancer patients	[154]
A prognostic miRNA panel template (PROMPT): miRNAs, miR-141, miR-144, miR-193b, miR-200a, miR-200b, miR-200c, miR-203, miR-210, miR-215, miR-365, miR-375, miR-429, miR-486-5p, miR-801, miR-1260, and miR-1274a	Associated with OS and RFS	Sensitivity: 77% Specificity: 75%	A total of 237 metastatic breast cancer patients	[155]
miR-21, miR-23b, miR-200c, and miR-190	An increase in the expression of miR-21, miR-23b, and miR-200c, accompanied by a decrease in miR-190 in relapsed patients, compared to the non-relapsed ones	Sensitivity: 71.4% Specificity 63.9%	A total of 49 relapsed and 84 non-relapsed localized breast cancer patients	[18]
For predicting treatment response				
miR-125b	Correlation between miR-125b and chemotherapeutic resistance	-	-	[156]
miR-155	miR-155 serum levels decreased after surgery and four cycles of chemotherapy	-	-	[157]
EV				
For diagnosis				
Exosomoal miR-142-5p, miR-320a, and miR-4433b-5p	For breast cancer diagnosis	Sensitivity: 93.33% Specificity: 68.75%	A total of 31 breast cancer patients	[158]
Exosomal miR-424, miR-423, miR-660, and let7-i	For breast cancer detection	Sensitivity: 98.6% Specificity: 100%	A total of 69 breast cancer patients and 40 healthy controls	[159]
Exosomal miR-188-3p, miR-500a-5p, and miR-501-5p in plasma; exosomal miR-188-3p, miR-501-3p, miR-502-3p, miR-532-3p, and miR-532-5p in serum	Upregulated in breast cancer patients	-	A total of 800 plasma and serum samples from breast cancer patients and healthy controls	[160]
let-7b-5p, miR-106a-5p, miR-19a-3p, miR-19b-3p, miR-25-3p, miR-425-5p, miR-451a, miR-92a-3p, miR-93-5p, and miR-16-5p	Upregulated in serum-derived exosomes in breast cancer patients, compared to controls	Specificity: 94.9% Sensitivity: 96.2%	A total of 32 pairs of breast cancer patients and healthy controls	[161]
Exosomal lncRNA *H19*	Exosomal levels of the lncRNA *H19* were significantly higher in breast cancer patients than healthy controls	Sensitivity: 87.0% Specificity: 70.6%	A total of 50 patients	[162]
Exosomal Hsp70	Increased levels of exosomal Hsp70 in breast cancer patients, compared to healthy donors	-	A total of 40 patients and 14 healthy volunteers	[163]
Claudin-7 and claudin-7/CD81 levels in EVs	Claudin-7 might be a universal marker for the early diagnosis of breast cancer	Sensitivity: 95% Specificity: 75.13%	A total of 60 breast cancer patients and 20 healthy volunteers	[164]
Seven proteins (fibronectin, focal adhesion kinase 1 (FAK), dual-specificity mitogen-activated protein kinase kinase 1, β-Actin, p90RSK_pT573, N-cadherin, and proto-oncogene c-RAF)	Distinguish patients (early patients accounted for nearly 70%) with breast cancer from healthy individuals	Sensitivity: 94% Specificity: 82%	A total of 27 patients and 22 healthy controls	[165]
EGFR in EV	Diagnosing breast cancer patients with different clinical stages (I–IV)	Sensitivity: 90%	n = 49: 6 healthy control, 5 benign tumor, and 38 malignant tumor, including 13 with stage I, 14 with stage II, 5 with age III, 2 with stage IV, 4 without stage information	[166]
Eight plasma EV protein markers (mucin-1, CA-125, carcinoembryonic antigen, HER2, EGFR, PSMA, EpCAM, and VEGF)	Distinguish among metastatic breast cancer, nonmetastatic breast cancer, and healthy donors	Overall accuracy: 91.1%	A total of 220 plasma samples from breast cancer patients	[167]
Exosomal AnxA2	Higher expression of serum exosomal AnxA2 in breast cancer patients compared to non-cancer females; high expression of exosomal AnxA2 levels in was significantly associated with poor overall survival and poor disease-free survival	-	A total of 169 breast cancer patients and 68 non-cancer females	[168]
γ-glutamyltransferase 1 in EVs	Patients with breast cancer had enhanced γ-glutamyltransferase 1 detection signals than those of healthy donors	-	Patients with breast cancer (five cases) and healthy donors (five cases)	[169]
For prognosis				
miR-21 and miR-105	miR-21 and miR-105 were overexpressed in metastatic patients, compared to non-metastatic ones, as well as controls	-	A total of 53 patients	[170]
Exosomal miR-30b, miR-328, and miR-423	Predicted pCR	-	A total of 20 breast cancer patients	[171]
Heat shock protein 70 in small EVs	Elevated in patients with recurrence or metastasis	-	27 patients; Serum of 40 breast cancer patients	[165,172]
For predicting treatment response				
Exosomal mRNAs encoding TK1 and CDK9	Elevated exosomal levels of mRNAs encoding TK1 and CDK9 were associated with poor clinical response to the CDK4/CDK6 inhibitor palbociclib	-	-	[173]
lncRNA *HOTAIR*	Possible predictor of response to chemotherapy and tamoxifen treatment	-	A total of 15 breast cancer patients treated surgically, 15 healthy individuals, 25 patients received neoadjuvant chemotherapy before surgery, and 25 patients received tamoxifen hormone treatment after surgery	[174]
ANXA6 in plasma EVs	Reflect treatment response of neo-adjuvant treatment	-	-	[175]
Protein				
CCN1	For early cancer detection	Specificity: 99.0% Sensitivity: 80.0%	A total of 544 patients with breast cancer and 427 healthy controls	[176]

**Table 2:** Detection techniques for liquid biopsy.

Detection Techniques	Target	Advantages	References
For CTC Detection			
CellSearch^®^	CTCs immunoisolation by positive selection targeting EpCAM	Gold standard and the only technique approved by the FDA for the isolation and detection of CTCs in metastatic breast, prostate, and colon cancer	[37,190]
Adnatest (QIAGEN^®^)	A combination of antibodies conjugated with magnetic beads for selecting tumor and epithelial markers and an RT-PCR for detecting breast cancer mRNAs biomarkers	Isolate CTCs in the breast cancer neoadjuvant setting	[191]
CTC-iChip	a digital RNA signature	For CTC isolation and detection in early and metastatic breast cancer patients	[192]
Nanotube-CTC-chip	Breast cancer-specific antibodies, such as anti-EpCAM and anti-her2	Identify CTCs in the 100% of the studied breast cancer peripheral blood samples	[193,194]
AFM chip	EpCAM, CK19, CD45, and DAPI	Highly efficient at rapidly capturing CTCs from cancer patients’ whole blood, without requiring extra equipment	[195]
For cfDNA detection			
The Oncomine Breast cfDNA (Thermofisher, Waltham, MA, USA) test	DNA	Detect mutations in a limited number of genes from breast cancer patients	[196]
dPCR	cfDNA: HER2	Could be used as a companion diagnostic tool to detect plasma HER2 status	[197]
For ctDNA detection			
ddPCR and the BEAMing technology	*PIK3CA* mutations in plasma ctDNA from advanced breast cancer patients	Allow absolute quantification of allele frequencies as low as 0.01%	[198,199,200]
*PIK3CA* RGQ PCR Kit	11 mutations in the *PIK3CA* gene from patients with advanced or metastatic breast cancer	May help doctors identify breast cancer patients who should be treated with PIQRAY^®^	[147]
NGS-based ctDNA test, Signatera™	ctDNA	For the detection of MRD after surgery and earlier detection of disease recurrence	[73]
TARDIS of ctDNA	Multiple tumor mutations in ctDNA	Highly sensitive method combining a targeted linear pre-amplification, followed by unique molecular identifiers (UMIs) ligation, targeted exponential PCR, and ultra-deep sequencing	[89]
SiMSen-Seq assay	*PIK3CA* mutations in ctDNA	Allows detection of extremely rare variant alleles at <0.1% frequency and shows advantageous concordance with the tissue analyses	[201]
INtegration of VAriant Reads (INVAR)	ctDNA detection; up to a thousand loci for mutations	As little as one mutant molecule per 100,000 can be detected, thus significantly increasing the ctDNA detection sensitivity	[202]
For miRNA detection			
SERS with SMGAPs	miR-21 and miR-155	SERS gives information for trace amount of material	[203]
For protein detection			
Localized fluorescent-imaging method	Multiple proteins on individual EVs	Enables the detection of multiple proteins on individual EVs	[204]
High-resolution flow cytometry	Proteins on EVs	Improve reporting and reliability of single EV flow cytometry experiments	[205]
Microfluidic devices	Proteins on EVs	Achieve higher specificity and sensitivity	[206]
The aptasensor method	Proteins on EVs	Pre-separation of EVs is not needed, the total detection time is short (within 3 h), and it has a low cost (less than $1)	[167]
